# A Cross-Sectional Analysis of Body Composition Among Healthy Elderly From the European NU-AGE Study: Sex and Country Specific Features

**DOI:** 10.3389/fphys.2018.01693

**Published:** 2018-11-30

**Authors:** Aurelia Santoro, Alberto Bazzocchi, Giulia Guidarelli, Rita Ostan, Enrico Giampieri, Daniele Mercatelli, Maria Scurti, Agnes Berendsen, Olga Surala, Amy Jennings, Nathalie Meunier, Elodie Caumon, Rachel Gillings, Fawzi Kadi, Frederic Capel, Kevin D. Cashman, Barbara Pietruszka, Edith J. M. Feskens, Lisette C. P. G. M. De Groot, Giuseppe Battista, Stefano Salvioli, Claudio Franceschi

**Affiliations:** ^1^Department of Experimental, Diagnostic and Specialty Medicine, Alma Mater Studiorum, University of Bologna, Bologna, Italy; ^2^C.I.G. Interdepartmental Centre “L. Galvani”, Alma Mater Studiorum, University of Bologna, Bologna, Italy; ^3^IRCCS Istituto Ortopedico Rizzoli, Bologna, Italy; ^4^Department of Physics, Alma Mater Studiorum, University of Bologna, Bologna, Italy; ^5^Institute of Neurological Sciences (IRCCS), Bologna, Italy; ^6^Division of Human Nutrition and Health, Wageningen University and Research, Wageningen, Netherlands; ^7^Department of Human Nutrition, Warsaw University of Life Sciences - SGGW, Warsaw, Poland; ^8^Norwich Medical School, University of East Anglia, Norwich, United Kingdom; ^9^CHU Clermont-Ferrand, Clermont-Ferrand, France; ^10^School of Health and Medical Sciences, Örebro University, Örebro, Sweden; ^11^Unité de Nutrition Humaine, INRA, Université Clermont Auvergne, Clermont-Ferrand, France; ^12^Cork Centre for Vitamin D and Nutrition Research, School of Food and Nutritional Sciences, University College Cork, Cork, Ireland

**Keywords:** body composition, DXA, elderly, sex, Europe, fat, lean and bone mass

## Abstract

Body composition (BC) is an emerging important factor for the characterization of metabolic status. The assessment of BC has been studied in various populations and diseases such as obesity, diabetes, endocrine diseases as well as physiological and paraphysiological conditions such as growth and aging processes, and physical training. A gold standard technique for the assessment of human BC at molecular level is represented by dual-energy X-ray absorptiometry (DXA), which is able to precisely assess the body mass (and areal bone mineral density-aBMD) on a regional and whole-body basis. For the first time, within the framework of the NU-AGE project, BC has been assessed by means of a whole-body DXA scan in 1121 sex-balanced free-living, apparently healthy older adults aged 65–79 years enrolled in 5 European countries (Italy, France, United Kingdom, Netherlands, and Poland). The aim of this analysis is to provide a complete profile of BC in healthy elderly participants from five European countries and to investigate country- and sex-related differences by state-of-the-art DXA technology. To compare BC data collected in different centers, specific indexes and ratios have been used. Non-parametric statistical tests showed sex-specific significant differences in certain BC parameters. In particular, women have higher fat mass (FM) (Fat/Lean mass ratio: by 67%, *p* < 2.2e-16) and lower lean mass (Lean Mass index: by -18%, *p* < 2.2e-16) than men. On the other hand, men have higher android FM than women (Android/gynoid FM ratio: by 56%, *p* < 2.2e-16). Interesting differences also emerged among countries. Polish elderly have higher FM (Fat/Lean mass ratio: by 52%, *p* < 2.2e-16) and lower lean mass (Skeletal Mass index: by -23%, *p* < 2.2e-16) than elderly from the other four countries. At variance, French elderly show lower FM (Fat/Lean mass ratio: by -34%, *p* < 2.2e-16) and higher lean mass (Skeletal Mass index: by 18%, *p* < 2.2e-16). Moreover, five BC profiles in women and six in men have been identified by a cluster analysis based on BC parameters. Finally, these data can serve as reference for normative average and variability of BC in the elderly populations across Europe.

## Introduction

Changes in body composition (BC) are associated with aging, wherein loss of muscle mass and increase in total fat mass (FM) occurs. In general, there is a decrease in subcutaneous FM, whereas visceral fat, liver fat, and muscle fat infiltration tend to increase with age. Monitoring weight among older adults is important as changes in weight may reflect declining health (Reinders et al., 2017). Alterations in body weight have been associated with a decline in health status among elderly. Previous studies have shown that weight loss, weight gain, and weight cycling are associated with higher mortality risk, with strongest associations showed for weight loss (Cheng et al., 2015). In addition, unintentional weight loss is associated with increased risk of incident mobility impairment (Murphy et al., 2014). However, the increase in total FM and the loss of lean mass are often independent from changes in weight and thus in body mass index (BMI). While BMI is a simple yet valid tool for assessing overall adiposity at population levels, it is subject to obvious limitations when quantifying individuals’ body fat contents (Prentice and Jebb, 2001; Zong et al., 2017). Indeed, BMI does not reflect the distribution of body fat, which is clearly associated with the development of chronic diseases and mortality (Carmienke et al., 2013; Tchkonia et al., 2013; Rost et al., 2018). Trunk fat has been linked to metabolic abnormalities at various BMI levels (Bjorntorp, 1991; Bosy-Westphal et al., 2015), and several studies have reported a positive association between visceral and neck adipose tissue and incidence of cardiovascular diseases (Arsenault et al., 2012; Britton et al., 2013; Torriani et al., 2014). Conversely, the accumulation of leg fat is associated with a largely favorable metabolic profile (Van Pelt et al., 2005; Wu et al., 2010; Zhang et al., 2013). Previous studies have also shown that preserved lean muscle mass with scarce muscle fat infiltration is associated with improved physical function and gait speed in general older populations (Beavers et al., 2013; Reinders et al., 2015). It has also been shown that increased muscle fat infiltration is associated with higher mortality risk (Miljkovic et al., 2015; Reinders et al., 2016).

Dual-energy X-ray absorptiometry (DXA) has gained interest and acceptance as a reference method for the assessment of human BC (Bazzocchi et al., 2013; Guglielmi et al., 2016), due to its relatively low cost, fast acquisition time and low radiation exposure, as compared to other available techniques (Hangartner et al., 2013; Bazzocchi et al., 2016; Lewiecki et al., 2016; Guerri et al., 2018). DXA measurements are based on a 3-compartment model that can be simplified into FM, non-bone lean mass (LM) and bone mineral content (BMC). This technique is able to assess the body masses and bone mineral density (BMD) on a regional and whole- body basis. All these advantages and the predisposition of the more recent DXA technologies to BC analysis make this densitometric method suitable for clinical use and longitudinal studies at all stages of life, from children to elderly (Bazzocchi et al., 2014, 2016; Diano et al., 2017). BC values can be considered among the most variable to be collected and analyzed, since the differences between human populations, countries and sex are remarkable (Kelly et al., 2009; Hinton et al., 2017). However, some parameters and indexes as measured with different techniques in the analysis of BC have been proposed for collection and comparative evaluations among healthy and unhealthy populations (Bazzocchi et al., 2016). DXA measures of adiposity and lean mass include Fat Mass index (FMI: total FM/height^2^); Visceral Adipose Tissue (VAT); Subcutaneous Adipose Tissue (SAT); android to gynoid FM ratio (A/G FM), trunk to leg FM ratio (T/L FM); Lean Mass index (LMI: total LM/height^2^); Appendicular Lean Mass (ALM: arms LM + legs LM) and the corresponding indexes standardized to height and weight called Appendicular Lean Mass index (ALMI: ALM/height^2^) and Skeletal Muscle Mass index (SMI: ALM/total weight), respectively (Petak et al., 2013). Dividing whole-body and regional BC parameters by squared height or total weight is fundamental for comparison among participants independently from their size (Heymsfield et al., 2007).

Nowadays, data on normal BC in the elderly are almost completely missing or partial. Thus, efforts in the definition of a normative database are highly desirable. To the best of our knowledge no study to date has evaluated BC parameters by DXA scan among elderly populations in Europe. Within the framework of the NU-AGE project a whole-body DXA scan has been performed in 1121 sex-balanced free-living, apparently healthy older adults aged 65–79 years enrolled in 5 European countries (Italy, France, United Kingdom, Netherlands, and Poland) (Santoro et al., 2014). Specifically, the aim of the current study is to investigate sex- and country-related differences among relatively healthy older adults from five different European countries using several BC parameters assessed by DXA in the NU-AGE project and to build specific BC profiles by a cluster analysis.

## Materials and Methods

### Study Design and Population

NU-AGE^1^ is a 1-year, multicenter, randomized, single-blind, controlled trial (registered with ClinicalTrials.gov, NCT01754012) carried out in five European centers located in France (Clermont-Ferrand), Italy (Bologna), Netherlands (Wageningen), Poland (Warsaw), and the United Kingdom (Norwich) (Santoro et al., 2014). The recruitment of participants has been described in detail previously (Berendsen et al., 2014; Santoro et al., 2014). Briefly, 2668 volunteers from the community aged 65–79 years, free of major overt chronic diseases for at least 2 years (i.e., cancer, severe organ disease), living independently, and free of dementia, were recruited from July 2012 to January 2014 to participate in the baseline assessment. At enrollment, exclusion criteria included severe heart diseases, type 1 and insulin-treated type 2 diabetes, chronic use of corticosteroids, recent use of antibiotics, change in habitual medication use, frailty (Fried et al., 2001), malnutrition (BMI < 18.5 kg/m^2^ or 10% weight loss within 6 months), or food allergy/intolerance requiring special diets. Of the 2668 participants, 1512 were screened for inclusion and 1296 were eligible to participate in the NU-AGE trial. In this study, 1121 participants underwent a whole-body, spine and hip DXA scans, at baseline, and were included from the NU-AGE study cohort. Written informed consent was collected from all participants prior to their inclusion in the study, in accordance with the Declaration of Helsinki (The World Medical Association Inc., 2018. DECLARATION OF HELSINKI Ethical Principles for Medical Research Involving Human Subjects). NU-AGE was approved by the Ethics Committee of the coordinator center: the Independent Ethics Committee of the S. Orsola-Malpighi Hospital Bologna (Italy), and by the local/national Ethics Committees of all the other four recruiting centers: the South-East 6 Person Protection Committee (France), the Wageningen University Medical Ethics Committee (Netherlands), the National Research Ethics Committee–East of England (United Kingdom), and the Bioethics Committee of the Polish National Food and Nutrition Institute (Poland).

### Assessment of Body Composition

A whole-body DXA scan was performed to measure total and regional BC using the following fan-beam densitometers in each recruiting center: Discovery QDR, Hologic Inc., Bedford, MA, United States – software version 3; BMD Coefficient of Variation (CV): ≤1.2% (Clermont-Ferrand, France); Lunar iDXA, GE Healthcare, Madison, WI, United States – enCORETM 2011 software version 13.6; BMD CV: ≤1.0% (Bologna, Italy); Lunar Prodigy, GE Healthcare, Madison, WI, United States – enCORETM 2011 software version 13.6 (BMD CV: ≤1.1% in Wageningen, Netherlands and BMD CV: ≤1.0% Warsaw, Poland); and Discovery Wi, Hologic Inc., Bedford, MA, United States, BMD CV: ≤1.1% (Norwich, United Kingdom). The scanners followed standard Quality Control procedures and they were calibrated daily using a standard calibration block supplied by the manufacturers. DXA scans were performed by trained technicians according to state-of-the-art technique and manufacturers recommendation. All metal items were removed before densitometry. Participants were placed in a supine position with arms at sides slightly separated from the trunk and correctly centered on the scanning field. Region of interests were defined by the analytical program including six different corporeal regions: Total body, trunk, upper limbs, lower limbs, android region (a portion of the abdomen included between the line joining the two superior iliac crests and extended cranially up to the 20% of the distance between this line and the chin), and gynoid region (a portion of legs from the femoral great trochanter, directed caudally up to a distance double of the android region). Android and gynoid regions were not defined by the densitometer used in United Kingdom. For each region, DXA scanned the weight (in g) of total fat, lean and bone mass: whole body FM, non-bone whole body lean mass (LM), and bone mineral content (BMC). The relationship between parameters derived from the different DXA machines was investigated using specific reliable indexes. In particular, total body FM/LM (a), Fat Mass index (FMI, whole body fat mass/height^2^) (b), Lean Mass index (LMI, whole body lean mass/height^2^) (c), android/gynoid FM (d), android FM/LM (e), Appendicular Lean Mass index (ALMI, lean mass from arms plus legs/height^2^) (f), and Skeletal Mass index (SMI, lean mass from arms plus legs/weight) (g) were considered as the pivotal parameters of BC, in terms of general mass balance (a, b, c), central/peripheral distribution of FM (d), central abdominal distribution (e), low muscle mass (f, g), respectively (Petak et al., 2013; Bazzocchi et al., 2016). Moreover, total body (tb), femoral neck (fn) and spinal BMD and *T*-score were also considered as parameters of bone health. *T*-score represents a comparison of a patient’s BMD to that of a healthy 30-year-old, with values ranging from: -1 or higher for normality, from -1 and -2.5 for osteopenia and -2.5 or lower for osteoporosis.

### Data Collection

Adherence to the NU-AGE diet has been calculated by the NU-AGE index (Berendsen et al., unpublished). The NU-AGE index is a 160 points scale comprising recommendations of minimum amounts to consume for fruits, vegetables, legumes, low-fat dairy, low-fat cheese, fish, low-fat meat and poultry, nuts, olive oil, fluids, and vitamin D_3_ (from a supplement), of minimum and maximum intake frequencies, for whole grains and eggs, and recommendations to limit, alcohol, salt and sweets. In this paper we used the NU-AGE index transformed in percentage and scaled 0–100 (0: no adherence, 100: fully adherent).

Data on educational level (level and years), physical activity [Physical activity scale for the elderly, PASE (Washburn et al., 1993)], and medical history (use of drugs for hypertension [yes/no], use of drugs for diabetes [yes/no], use of drugs for hypercholesterolemia [yes/no], use of vitamin D supplementation [yes/no], use of calcium supplementation [yes/no]) were obtained by means of questionnaires. Height was measured with a stadiometer to the nearest 0.1 cm. Weight was measured to the nearest 0.1 kg with a calibrated scale while wearing light clothes. BMI was calculated as weight [kg]/height[m]^2^. Calorie intake was calculated by mean of the 7 days food record completed by the participants at baseline. Handgrip strength test was performed by standardized procedures using Jamar handheld dynamometer (Patterson Medical, Warrenville, IL, United States). Blood pressure was measured using automated and calibrated electronic blood pressure monitors [(United Kingdom: Omron HEM-7117-E, Milton Keynes, United Kingdom; France: 2 devices were used. From July 2012 (first NUAGE volunteer included) to 31/08/2014: Dynamap Weleh Allyn, IMEDA – From 01/09/2014 to end of the project OMRON M6W, Santé France, France; Italy: Omron, M2 compact, Milano, Italy; Dinamap Pro 100, KP Medical, Houten, Netherlands; Omron M2 Basic HEM-7116-E8(v) Omron Healthcare Co. Ltd., Kyoto, Japan]. All measures were taken by trained research assistants.

Glycated hemoglobin was measured on fresh blood in each recruiting centers by standard methods. Plasma total, HDL and LDL cholesterol (mg/dL) and triglycerides (mg/dL) were measured on a konelab system and reagents were from Thermo Scientific (Asnières sur Seine, France).

Concentrations of total 25-hydroxyvitamin D [25(OH)D] [i.e., 25(OH)D2 plus 25(OH)D3] and parathyroid hormone (PTH) in all serum samples were measured at the laboratory of the Cork Centre for Vitamin D and Nutrition Research. 25(OH)D was measured by a modified version of the LC-MS/MS method that has been described in detail elsewhere (Cashman et al., 2013) and is certified by the Centers for Disease Control and Prevention’s (CDC) Vitamin D Standardization Certification Program (Vitamin D Standardization-Certification Program [VDSCP], 2018: List of Certified Participants). PTH was measured with an ELISA kit (intact PTH; MD Biosciences Inc.). Intra-assay and inter- assay CVs were 3.0 and 5.1%, respectively (at a concentration of 47.7 and 52.6 pg/ml, respectively). All the other biochemical analyses glucose (mmol/L), insulin (mcU/mL), albumin (g/L), and creatinine (mmol/L), were measured on frozen blood and frozen urine (urea) in a centralized center with standard methodologies.

### Statistical Methods

According to Shapiro–Wilk test for Normality (*p* < 0.01) we decided to use non-parametric statistical tests. R studio (Version ‘1.0.136’ for Windows) was used for the analysis and results are reported as mean and standard deviation (±SD). Data were analyzed by Mann–Whitney and Kruskal–Wallis tests to determine differences between men and women and between the five countries. Pairwise comparison method was applied to test differences between all pairs of country. A type I error of 0.05 (*p*-value) in two-tailed tests was considered significant. Due to multiple testing of the variables, the Benjamini–Hochberg correction was applied (q-value). According to the International Society of Clinical Densitometry (ISCD) guidelines (Baim et al., 2005), the least significant change in BMD that can be recognized with 95% confidence is 2.77 × CV. In this study, a BMD difference among countries higher than 3.32% [the highest CV percentage (1.2) × 2.77] has been considered significant. Furthermore, BMI is widely used as an index of relative weight but its relation with BC is controversial, indeed BMI cannot distinguish fat and lean masses. Therefore, we decided to perform a hierarchical cluster analysis to detect different groups based on BC’s parameters together with BMI. There are two main methods: a hierarchical and non-hierarchical cluster analysis. The present paper focuses on hierarchical clustering which does not require an initial cluster number specification (Chalise et al., 2014). Hierarchical clustering is a statistical classification technique where data (participants) are grouped together into homogeneous groups called ’clusters’ one at a time in a series of sequential steps. The aim is increasing within-group homogeneity and between-groups heterogeneity, meaning that people within the same cluster have similar BC but different from people in the other clusters. Men and women were separately investigated in this analysis.

## Results

### Anthropometric, Nutritional, Physical and Body Composition Characteristics of NU-AGE Study Participants by Sex

1121 NU-AGE participants, 620 women (55%) and 501 men (45%), who completed the BC assessment at baseline were included in this study. As shown in Table 1, anthropometric measures, education, NU-AGE index and calorie intake, physical functioning and BC parameters considered are significantly different between men and women (Table 1). All subsequent analyses were then conducted separately by sex. Men have higher height, weight, BMI, waist circumference and waist-to-hip ratio, calorie intake, PASE score and handgrip strength than women. Women have significantly higher FM parameters than men in terms of FM, FMI, FM/LM and android FM/LM, and lower android/gynoid FM, while men have significantly higher lean mass parameters than women in terms of LM, ALMI, LMI, SMI and also higher bone content parameters in terms of tbBMC, tbBMD, tbT-score, L1-L4 *T*-score, L1-L4 BMD, fn T-score and fn BMD.

**Table 1:** Characteristics of the NU-AGE participants (*N* = 1,121) by sex.

Characteristics	Women	Men	*p*-value	*q*-value
	*n* = 620	*n* = 501		
Age (*years*)	70.7 ± 3.9	71.0 ± 4.1	NS	NS
Weight *(kg)*	67.7 ± 11.2	80.6 ± 12.6	<2.2e-16	<2.2e-16
Height *(cm)*	160.0 ± 6.7	173.0 ± 6.4	<2.2e-16	<2.2e-16
BMI (*kg/m^2^*)	26.5 ± 4.1	26.9 ± 3.7	1.16e-02	2.73e-02
Waist circumference (*cm*)	86.9 ± 10.8	96.7 ± 11.1	<2.2e-16	<2.2e-16
Hip circumference (*cm*)	103.3 ± 9.1	101.5 ± 7.6	1.32e-03	3.54e-03
Waist to hip circumference ratio	0.85 ± 0.31	0.95 ± 0.06	<2.2e-16	<2.2e-16
**Education**				
Primary school, *N* (%)	25 (4.0)	12 (2.4)	NS	NS
Low secondary school, *N* (%)	71 (11.5)	72 (14.4)		
Up secondary school, *N* (%)	238 (38.4)	195 (38.9)		
College, *N* (%)	286 (46.1)	222 (44.3)		
Education (years)	12.4 ± 3.4	13.0 ± 3.8	2.15e-02	NS
**Diet assessment**				
Adherence to NU-AGE diet	52.5 ± 10.3	50.0 ± 9.3	6.80e-05	2.16e-04
Calorie Intake (*kcal*)	1680.9 ± 327.8	2123.3 ± 445.0	<2.2e-16	<2.2e-16
**Physical functioning**				
Hand grip strength (*kg*)	25.2 ± 5.5	39.6 ± 7.0	<2.2e-16	<2.2e-16
PASE Score	127.8 ± 48.9	140.9 ± 59.5	3.53e-04	1.01e-03
**Body composition parameters**				
FM (*kg*)	26.2 ± 8.06	22.0 ± 8.37	<2.2e-16	<2.2e-16
FMI (*kg/m^2^*)	10.3 ± 3.16	7.35 ± 2.74	<2.2e-16	<2.2e-16
FM/LM	0.65 ± 0.19	0.39 ± 0.14	<2.2e-16	<2.2e-16
LM (*kg*)	40.3 ± 4.97	57.1 ± 6.71	<2.2e-16	<2.2e-16
LMI (*kg/m^2^*)	15.7 ± 1.53	19.1 ± 1.80	<2.2e-16	<2.2e-16
ALMI (*kg/m^2^*)	6.56 ± 0.77	8.47 ± 0.87	<2.2e-16	<2.2e-16
SMI	0.25 ± 0.03	0.32 ± 0.04	<2.2e-16	<2.2e-16
tbT-score	–0.82 ± 1.2	–0.19 ± 1.2	<2.2e-16	<2.2e-16
tbBMC (*g*)	2092.5 ± 357	2947.8 ± 483	<2.2e-16	<2.2e-16
tbBMD (g/cm^2^)	1.03 ± 0.11	1.19 ± 0.11	<2.2e-16	<2.2e-16
Android/Gynoid FM^∗^	0.50 ± 0.15	0.78 ± 0.21	<2.2e-16	<2.2e-16
Android FM/LM^∗^	0.79 ± 0.30	0.61 ± 0.25	2.70e-16	2.82e-15
L1-L4 BMD (g/cm^2^)^Δ^	1.0 ± 0.17	1.17 ± 0.2	<2.2e-16	<2.2e-16
L1-L4 *T*-score^Δ^	–1.0 ± 1.4	–0.11 ± 1.65	2.74e-05	9.53e-05
fnBMD (g/cm^2^)^Δ^	0.78 ± 0.12	0.88 ± 0.14	<2.2e-16	<2.2e-16
fnT-score^Δ^	–1.36 ± 0.93	–1.07 ± 0.9	<2.2e-16	<2.2e-16

*Anthropometric, education, diet, physical and body composition parameters are described in women and men.*

*BMI, body mass index; FM, fat mass; FMI, fat mass index; LM, lean mass; LMI, non-bone lean mass index; ALMI, non-bone appendicular lean mass index; SMI, skeletal mass index; tbBMC, total body bone mineral content; tbBMD, total body bone mineral density; tbT-score, total body T-score; L1-L4 BMD, spinal BMD; L1-L4 T-score, spinal T-score; fnBMD, femoral neck bone mineral density fnT-score, femoral neck T-score; Values are means ± SDs, unless otherwise stated. NS, not statistically significant. ^∗^(F = 474, M = 416); Δ (F = 387, M = 298); p-value (Mann–Whitney and Kruskal–Wallis tests); q-value (Benjamini–Hochberg multiple testing correction).*

### Anthropometric, Nutritional, Physical and Body Composition Characteristics of NU-AGE Study Participants by Country

Characteristics of the participants described in Table 1 were also analyzed separately according to country of origin by the Kruskal–Wallis test (Table 2). As reported in Table 2 French participants have the lowest weight, BMI, hip and waist circumference, while Polish participants have the highest ones, with the exception of hip circumference that is slightly higher in English participants. French participants have also the highest adherence to the NU-AGE diet at baseline and the highest calorie intake, while Dutch participants have the lowest adherence and Italian participants the lowest calorie intake. English participants have the highest handgrip strength value and PASE score, while Polish participants have the lowest handgrip strength, and Italian participants have the lowest PASE score (Table 2). As far as the BC parameters, Polish participants have the highest FM parameters in terms of FM, FMI, FM/LM, and android FM/LM, while French participants have the lowest ones, including also android/gynoid FM. The lean mass parameters reach the highest values in French participants (LM, ALMI, LMI, and SMI) while the lowest ones are found in Italian (LM and LMI) and Polish participants (ALMI and SMI). Bone mass parameters reach the highest values in Dutch participants (tbBMC, tbT-score, and tbBMD) and the lowest ones in English participants (L1-L4 BMD and neck fnBMD) (Table 2).

**Table 2:** Characteristics of the NU-AGE participants by country of origin (*N* = 1,121).

Characteristics	Italy	Poland	United Kingdom	France	Netherlands	*p*-value	*q*-value
	*n* = 236	*n* = 222	*n* = 246	*n* = 184	*n = 233*		
Age (*years*)	71.7 ± 3.8	71.3 ± 3.8	70.1 ± 3.9	70.1 ± 3.8	71.0 ± 4.1	1.31e-06	5.33e-06
Female sex	119 (50.4)	127 (57.2)	154 (62.6)	91 (49.5)	129(55.1)	3.35e-02	NS
Weight (*kg*)	72.7 ± 12.7	75.7 ± 14.5	73.5 ± 13.5	70.0 ± 12.7	74.7 ± 13.4	1.65e-03	4.34e-03
Height (*cm*)	163.9 ± 9.4	163.9 ± 9.3	166.0 ± 9.0	166.0 ± 9.0	169.2 ± 8.2	8.59e-10	2.86e-09
BMI (*kg/m^2^*)	27.0 ± 3.8	28.0 ± 4.1	26.6 ± 3.9	25.4 ± 3.4	26.0 ± 3.6	1.45e-11	1.05e-10
Waist circumference (*cm*)	92.8 ± 11.4	93.3 ± 11.8	91.4 ± 12.0	86.3 ± 11.4	91.6 ± 11.9	1.98e-08	1.03e-07
Hip circumference (*cm*)	101.4 ± 7.4	103.6 ± 8.7	104.7 ± 9.1	99.1 ± 8.5	103.2 ± 7.9	2.45e-12	1.10e-11
Waist to hip circumference ratio	0.9 ± 0.1	0.9 ± 0.1	0.9 ± 0.1	0.9 ± 0.1	0.9 ± 0.1	4.75e-09	
**Education**							
Primary school N, (%)	25 (10.6)	0 (0.0)	1 (0.4)	4 (2.2)	7 (3.0)	<2.2e-16	<2.2e-16
Low secondary school *N*, (%)	61 (25.9)	8 (3.6)	0 (0.0)	46 (25.0)	28 (12.0)		
Up secondary school *N*, (%)	94 (39.8)	41 (18.5)	88 (35.8)	60 (32.6)	149 (64.0)		
College *N*, (%)	56 (23.7)	172 (77.5)	157 (63.8)	74 (40.2)	49 (21.0)		
Education (years)	11.2 ± 4.2	15.4 ± 2.7	11.8 ± 1.7	12.5 ± 3.7	12.3 ± 3.7	<2.2e-16	<2.2e-16
**Diet assessment**							
Adherence to NU-AGE diet	52.8 ± 9.5	52.7 ± 10.1	50.5 ± 8.8	55.9 ± 9.1	46.3 ± 9.8	<2.2e-16	<2.2e-16
Calorie Intake (*kcal*)	1733 ± 376	1850 ± 518	1903 ± 389	2024 ± 482	1912 ± 405	1.60e-09	9.62e-09
**Physical functioning**							
Handgrip strength (*kg*)	31.1 ± 9.7	30.4 ± 9.9	34.8 ± 9.1	31.1 ± 8.8	30.5 ± 9.3	3.53e-07	1.52e-06
Women	23.5 ± 5.3	23.7 ± 4.6	29.7 ± 5.4	23.9 ± 4.1	23.8 ± 4.8	<2.2e-16	<2.2e-16
Men	38.9 ± 6.6	39.3 ± 7.7	43.4 ± 7.3	38.2 ± 5.8	38.6 ± 6.6	1.00e-05	3.56e-05
PASE score	114.5 ± 50.9	131.7 ± 63.6	151.2 ± 53.0	134.9 ± 50.6	137.2 ± 52.6	2.52e-13	2.31e-12
**Body composition parameters**							
FM (*kg*)	26.2 ± 7.4	28.0 ± 9.1	23.3 ± 7.9	20.4 ± 7.4	23.2 ± 8.5	<2.2e-16	<2.2e-16
FMI (*kg/m^2^*)	9.9 ± 3.0	10.5 ± 3.5	8.5 ± 3.1	7.5 ± 2.9	8.2 ± 3.1	<2.2e-16	<2.2e-16
FM/LM	0.60 ± 0.19	0.64 ± 0.24	0.50 ± 0.18	0.42 ± 0.17	0.49 ± 0.20	<2.2e-16	<2.2e-16
LM (*kg*)	45.0 ± 8.7	45.6 ± 10.3	48.8 ± 10.4	50.8 ± 10.6	49.2 ± 9.9	7.45e-11	4.96e-10
LMI (*kg/m^2^*)	16.6 ± 2.0	16.7 ± 2.3	17.6 ± 2.5	18.3 ± 2.3	17.0 ± 2.2	1.68e-12	1.39e-11
ALMI (*kg/m^2^*)	7.4 ± 1.2	7.1 ± 1.1	7.5 ± 1.3	7.9 ± 1.3	7.2 ± 1.2	3.79e-09	2.13e-08
SMI	0.28 ± 0.03	0.26 ± 0.04	0.28 ± 0.05	0.32 ± 0.05	0.28 ± 0.05	<2.2e-16	<2.2e-16
tbT-score	–0.69 ± 1.15	–0.19 ± 1.25	–0.92 ± 1.23	–0.58 ± 1.23	–0.29 ± 1.21	3.90e-12	3.17e-11
tbBMC (*g*)	2463 ± 597	2610 ± 615	2233 ± 494	2327 ± 489	2729 ± 621	<2.2e-16	<2.2e-16
tbBMD (*g/cm^2^*)	1.07 ± 0.15	1.15 ± 0.12	1.06 ± 0.13	1.11 ± 0.12	1.14 ± 0.12	<2.2e-16	<2.2e-16
Android/Gynoid FM^∗^	0.65 ± 0.22	0.63 ± 0.21	–	0.54 ± 0.20	0.67 ± 0.24	9.06e-08	4.25e-07
Android FM/LM^∗^	0.77 ± 0.28	0.85 ± 0.28	–	0.45 ± 0.18	0.70 ± 0.25	<2.2e-16	<2.2e-16
L1-L4 *T*-score^Δ^	–0.84 ± 1.38	–0.36 ± 1.78	–0.63 ± 1.53	–	–	4.14e-02	NS
L1-L4 BMD (g/cm^2^)^Δ^	1.10 ± 0.17	1.15 ± 0.22	0.97 ± 0.18	–	–	<2.2e-16	<2.2e-16
fnT-score^Δ^	–1.39 ± 0.95	–1.19 ± 0.92	–1.12 ± 0.96	–	–	2.92e-02	NS
fnBMD (g/cm^2^)^Δ^	0.85 ± 0.13	0.89 ± 0.13	0.75 ± 0.12	–	–	<2.2e-16	<2.2e-16

*Anthropometric, education, diet, physical and body composition parameters are described in Italy, Poland, United Kingdom, France, and Netherlands.*

*BMI, body mass index; FM, fat mass; FMI, fat mass index; LM, lean mass; LMI, non-bone lean mass index; ALMI, non-bone appendicular lean mass index; SMI, skeletal mass index; tbBMC, total body bone mineral content; tbBMD, total body bone mineral density; tbT-score, total body T-score; L1-L4 BMD, spinal BMD; L1-L4 T-score, spinal T-score; fnBMD, femoral neck bone mineral density fnT-score, femoral neck T-score; Values are means ± SDs, unless otherwise stated. NS, not statistically significant.^∗^ (N = 875); Δ (N = 704); p-value (Mann–Whitney and Kruskal–Wallis tests); q-value (Benjamini–Hochberg multiple testing correction).*

For each parameter we also conducted a comparison by country and by sex. The results are summarized in Supplementary Figures S1–S4.

### Identification of Specific Body Composition Clusters

In order to identify specific BC profiles among the participants and the relative overlap with BMI, a cluster analysis was performed separately within women (*n* = 620) and men (*n* = 501) using the following ten BC parameters: FM, FMI, LM, LMI, ALMI, FM/LM, SMI, tbT-score, tbBMC, and tbBMD in combination with BMI. Five clusters for women and six clusters for men were identified. According to the mean value of BMI we named these clusters as: Normal Weight (NW; BMI = 21.4 kg/m^2^; *N* = 89), Overweight A (OWA; BMI = 25.1 kg/m^2^; *N* = 251), Overweight B (OWB; BMI = 26.6 kg/m^2^; *N* = 137), Low Obesity A (LOA; BMI = 31.5 kg/m^2^; *N* = 61), and Low Obesity B (LOB; BMI = 31.9 kg/m^2^; *N* = 82) in women (Table 3A) and Normal Weight (NW; BMI = 24.0 kg/m^2^; *N* = 122), Overweight A (OWA; BMI = 25.7 kg/m^2^; *N* = 20), Overweight B (OWB; BMI = 26.3 kg/m^2^; *N* = 233), Low Obesity A (LOA; BMI = 30.1 kg/m^2^; *N* = 34), Low Obesity B (LOB; BMI = 30.4 kg/m^2^; *N* = 80) and Moderate Obesity (MO; BMI = 35.5 kg/m^2^; *N* = 12) in men (Table 3B).

**Table 3A:** Body composition and BMI characteristics of the five groups identified by cluster analysis in women (*N* = 620).

Clusters	BMI	FM	FMI	LM	LMI	ALMI	FM/LM	SMI	tbT score	tbBMC	tbBMD
	(kg/m^2^)	(kg)	(kg/m^2^)	(kg)	(kg/m^2^)	(kg/m^2^)				(g)	(g/cm^2^)
**Normal weight**	21.4 ± 1.7	15.9 ± 3.4	6.1 ± 1.3	38.4 ± 3.1	14.9 ± 1.1	6.2 ± 0.6	0.4 ± 0.1	0.29 ± 0.03	–1.4 ± 1.0	1905.1 ± 230.9	1.0 ± 0.1
(NW; *n* = 89; 14.4%)											
**Overweight A**	25.1 ± 1.9	23.8 ± 4.0^1^	9.2 ± 1.5^1^	40.4 ± 4.1^1^	15.5 ± 1.1^1^	6.4 ± 0.5	0.6 ± 0.1^1^	0.26 ± 0.02^1^	–0.4 ± 1.0^1^	2190.4 ± 294.9^1^	1.1 ± 0.1^1^
(OWA; *n* = 251; 40.5%)											
**Overweight B**	26.6 ± 2.7	26.9 ± 5.9	10.9 ± 2.3	37.2 ± 3.7	15.1 ± 1.0	6.3 ± 0.6	0.7 ± 0.2	0.24 ± 0.02	–1.9 ± 0.8	1804.1 ± 249.5	0.9 ± 0.1
(OWB; *n* = 137; 22.1%)											
**Low Obesity A**	31.5 ± 4.1	32.7 ± 6.4^a^	12.9 ± 2.6^a^	47.1 ± 5.8^a^	18.6 ± 1.7^a^	8.0 ± 0.8^a^	0.7 ± 0.1^a^	0.25 ± 0.02^a^	–0.6 ± 1.5^a^	2133.4 ± 378.2^a^	1.1 ± 0.1^a^
(LOA; *n* = 61; 9.8%)											
**Low Obesity B**	31.9 ± 2.4	38.5 ± 5.4	14.9 ± 2.0	42.1 ± 4.4	16.2 ± 1.1	6.8 ± 0.6	0.9 ± 0.1	0.21 ± 0.02	0.2 ± 0.8	2454.4 ± 297.3	1.1 ± 0.1
(LOB; *n* = 82; 13.2%)											

*Values are expressed as mean values ± SD, unless otherwise indicated.*

*^*1*^Significant difference between OWA and OWB (^1^p < 0.0001).*

*^*a*^Significant difference between LOA and LOB (^*a*^p < 0.0001).*

*BMI, body mass index; FM, fat mass; FMI, fat mass index; LM, lean mass; LMI, non-bone lean mass index; ALMI, non-bone appendicular lean mass index; SMI, skeletal mass index; tbT-score, total body T-score; tbBMC, total body bone mineral content; tbBMD, total body bone mineral density.*

**Table 3B:** Body composition and BMI characteristics of the six groups identified by cluster analysis in men (*N* = 501).

Clusters	BMI	FM	FMI	LM	LMI	ALMI	FM/LM	SMI	tbT score	tbBMC	tbBMD
	(kg/m^2^)	(kg)	(kg/m^2^)	(kg)	(kg/m^2^)	(kg/m^2^)				(g)	(g/cm^2^)
**Normal weight**	24.0 ± 2.1	13.7 ± 4.2	4.6 ± 1.4	57.0 ± 5.5	19.2 ± 1.4	8.5 ± 0.7	0.2 ± 0.1	0.36 ± 0.03	–0.9 ± 1.0	2631.9 ± 418.8	1.1 ± 0.1
(NW; *n* = 122; 24.4%)											
**Overweight A**	25.7 ± 2.8	15.6 ± 5.3^1^	5.1 ± 1.6^1^	61.8 ± 6.6^1^	20.3 ± 1.3^1^	9.1 ± 0.6^1^	0.2 ± 0.1^1^	0.36 ± 0.03^1^	1.9 ± 0.6^1^	3576.2 ± 401.1^1^	1.4 ± 0.1^1^
(OWA; *n* = 20; 4.0%)											
**Overweight B**	26.3 ± 2.3	22.2 ± 5.3	7.5 ± 1.8	54.0 ± 5.3	18.2 ± 1.3	8.1 ± 0.7	0.4 ± 0.1	0.31 ± 0.02	–0.4 ± 0.9	2891.4 ± 331.9	1.2 ± 0.1
(OWB; *n* = 233; 46.5%)											
**Low Obesity A**	30.1 ± 1.6	23.3 ± 4.8^a^	7.8 ± 1.5^a^	65.8 ± 5.2^a^	22.0 ± 1.1^a^	9.8 ± 0.6^a^	0.4 ± 0.1^a^	0.32 ± 0.03^a^	–0.5 ± 0.9^a^	2791.9 ± 347.2^a^	1.6 ± 0.1^a^
(LOA; *n* = 34; 6.8%)											
**Low Obesity B**	30.4 ± 2.9	31.5 ± 5.4	10.3 ± 1.9	59.5 ± 5.8	19.3 ± 1.6	8.5 ± 0.8	0.5 ± 0.1	0.28 ± 0.02	0.7 ± 1.1	3391.6 ± 432.8	1.3 ± 0.1
(LOB; *n* = 80; 16.0%)											
**Moderate Obesity**	36.6 ± 2.9	42.4 ± 5.1	13.9 ± 1.5	67.3 ± 7.8	21.9 ± 2.0	8.5 ± 1.1	0.6 ± 0.1	0.26 ± 0.03	1.6 ± 1.2	3667.6 ± 637.3	1.3 ± 0.1
(MO; *n* = 12; 2.3%)											

*Values are expressed as mean values ± SD, unless otherwise indicated.*

*^*1*^Significant difference between OWA and OWB (^*1*^p < 0.0001).*

*^*a*^Significant difference between LOA and LOB (^*a*^p < 0.0001).*

*BMI, body mass index; FM, fat mass; FMI, fat mass index; LM, lean mass; LMI, non-bone lean mass index; ALMI, non-bone appendicular lean mass index; SMI, skeletal mass index; tbT-score, total body T-score; tbBMC, total body bone mineral content; tbBMD, total body bone mineral density.*

The majority of women were grouped in the two overweight clusters: 40.5% in OWA and 22,1% in OWB, NW represents the 14.4%, LOA 9.8% and LOB 13.2%.

Within the five groups identified in women, as the BMI increases there is a general increase in FM, based on FM, FMI, and FM/LM parameters, while lean and bone masses do not show a specific correlation with BMI. It is interesting to note that clusters with very similar BMI such as OWA (BMI = 25.1 kg/m^2^) and OWB (BMI = 26.6 kg/m^2^) and LOA (BMI = 31.5 kg/m^2^) and LOB (BMI = 31.9 kg/m^2^) have a very different BC in terms of fat, lean and bone masses. Comparing the two overweight groups, OWA with respect to OWB has lower FM in terms of FM, FMI and FM/LM, but higher lean mass in terms of LM LMI and SMI and bone mass in terms of tbT score, tbBMC and tbBMD. Comparing the two low obesity groups, LOA with respect to LOB has lower FM in terms of FM, FMI, and FM/LM and bone mass in terms of tbT score, tbBMC and tbBMD, but higher lean mass in terms of LM, LMI. ALMI and SMI (Table 3A).

As far as the six clusters identified in men the majority were grouped in the OWB clusters (46.5%) and in NW (24.3%), OWA represents the 4.0%, LOA 6.8%, LOB 16.0% and MO 2.4%.

Also in men as BMI increases there is a general increase in FM, based on FM, FMI, and FM/LM parameters, while lean and bone masses do not show a specific correlation with BMI (Table 3B).

As far as women, also in men the clusters with very similar BMI such as OWA (BMI = 25.7 kg/m^2^) and OWB (BMI = 26.3 kg/m^2^) and LOA (BMI = 30.1 kg/m^2^) and LOB (BMI = 30.4 kg/m^2^) have a very different BC in terms of fat, lean and bone masses. Comparing the two overweight groups, OWA with respect to OWB has lower FM in terms of FM, FMI, and FM/LM, but higher lean mass in terms of LM, LMI, ALMI, and SMI and bone mass in terms of T score, BMC and BMD. Comparing the two low obesity groups, LOA with respect to LOB has lower FM in terms of FM, FMI and FM/LM and bone mass in terms of tbT-score and tbBMC but higher and tbBMD and lean mass in terms of LM, LMI, ALMI, and SMI (Table 3B).

It is interesting to note that among the six clusters identified in men the twelve participants belonging to the MO group (BMI = 35.5 kg/m^2^) have the highest values for fat mass (FM = 42.4 kg; FMI = 13.9 kg/m^2^; FM/LM = 0.6) but also the highest values for some lean and bone mass parameters (LM = 67.3 kg; and BMC = 3667.6 g) (Table 3B).

Among the ten BC parameters used, SMI and tbBMD do not discriminate very much among the clusters both in men and women.

### Comparison of Metabolic Profile Across the Body Composition Clusters

In order to further investigate the characteristics of the BC profiles identified, we compared several metabolic parameters among the clusters in women and men (Table 4).

**Table 4A:** Metabolic profile across the five body composition clusters in women.

	Normal weight	Overweight A	Overweight B	Low obesity A	Low obesity B	*p*-value
	(*n* = 89)	(*n* = 251)	(*n* = 137)	(*n* = 61)	(*n* = 82)	
Adherence to NU-AGE diet	54.9 ± 11.0	52.1 ± 10.3	51.6 ± 9.0	54.1 ± 9.6	51.7 ± 11.7	NS
Calorie Intake (kcal)	1723.6 ± 286.6	1722.8 ± 332.2	1608.1 ± 308.1	1736.5 ± 358.1	1587.7 ± 330.8	2.38E-04
Adherence to NU-AGE diet	54.9 ± 11.0	52.1 ± 10.3	51.6 ± 9.0	54.1 ± 9.6	51.7 ± 11.7	NS
Calorie intake (kcal)	1723.6 ± 286.6	1722.8 ± 332.2	1608.1 ± 308.1	1736.5 ± 358.1	1587.7 ± 330.8	2.38E-04
Total cholesterol (mg/dL)	230.9 ± 40.3	220.5 ± 39.8	222.5 ± 37.2	217.3 ± 40.6	214.3 ± 39.9	NS
HDL (mg/dL)	76.3 ± 19.8	66.9 ± 39.8	66.1 ± 16.5	60.1 ± 14.8	59.6 ± 16.9	4.72e-09
LDL (mg/dL)	136.8 ± 39.6	132.7 ± 36.4	136.2 ± 33.7	137.3 ± 38.9	129.9 ± 35.0	NS
Triglycerides (mg/dL)	89.1 ± 31.9	104.4 ± 45.7	100.9 ± 36.3	99.5 ± 33.1	123.7 ± 55.0	3.02e-05
Glycated hemoglobin (%)	5.7 ± 0.3	5.7 ± 0.3	5.7 ± 0.4	5.8 ± 0.4	5.9 ± 0.4	7.117e-03
Glucose (mmol/L)	5.2 ± 0.7	5.4 ± 0.6	5.5 ± 0.7	5.8 ± 1.1	5.9 ± 0.9	7.27e-09
Insulin (mcU/mL)	6.1 ± 3.4	8.4 ± 5.7	7.8 ± 3.8	11.3 ± 6.4	12.3 ± 6.3	2.95e-16
HOMA IR	1.5 ± 0.9	2.1 ± 1.6	1.9 ± 1.0	3.0 ± 2.0	3.3 ± 1.8	<2.2e-16
HOMA β (%)	74.3 ± 37.1	90.9 ± 56.5	84.3 ± 46.6	100.3 ± 49.8	109.1 ± 61.2	6.58e-05
Urinary urea (g/24 h)	16.9 ± 4.5	17.9 ± 5.1	16.8 ± 4.7	20.0 ± 4.9	20.1 ± 5.2	9.56e-08
Albumin (g/L)	45.7 ± 4.4	45.5 ± 4.2	44.7 ± 3.6	45.9 ± 4.1	44.9 ± 3.2	NS
Creatinine (mmol/L)	68.6 ± 11.9	69.8 ± 12.5	67.8 ± 11.7	71.4 ± 12.5	70.5 ± 10.3	NS
25 (OH)D (ng/mL)	26.8 ± 10.2	25.7 ± 8.9	24.9 ± 10.6	23.8 ± 9.4	23.3 ± 7.5	NS
PTH (pg/mL)	46.7 ± 32.1	40.3 ± 24.0	47.4 ± 28.9	41.9 ± 22.2	43.9 ± 22.8	NS
Diastolic pressure (mmHg)	70.6 ± 8.9	72.9 ± 10.0	75.3 ± 10.4	77.2 ± 9.9	75.7 ± 8.1	5.418e-05
Systolic pressure (mmHg)	132.7 ± 21.3	136.3 ± 19.9	138.3 ± 21.8	138.9 ± 20.5	139.9 ± 20.6	NS
**Use of medicines/supplements**						
Statins (*n* = 155; %)	18.0	25.9	20.4	29.5	34.1	NS
Diabetics (*n* = 16; %)	1.1	2.4	0.7	4.9	6.1	NS
Hypertension (*n* = 265; %)	23.6	43.4	46.0	34.4	62.2	8.035e-06
Vitamin D (*n* = 139; %)	22.5	23.5	25.5	13.11	20.7	<2.2e-16
Calcium (*n* = 78; %)	11.2	12.7	13.9	3.3	18.3	<2.2e-16
**Physical functioning**						
Handgrip strength (kg)	25.0 ± 5.7	25.4 ± 5.4	24.1 ± 5.9	26.7 ± 6.1	25.4 ± 4.7	2.529e-02
PASE score	141.2 ± 43.7	132.2 ± 48.6	125.3 ± 46.4	127.9 ± 52.6	104.2 ± 48.7	4.588e-06

*Population based reference ranges for: Total Cholesterol: <200 mg/dL; HDL, (high density lipoprotein): >60 mg/dL; LDL (low density lipoprotein): <100 mg/dL; Triglycerides: <150 mg/d; glycated hemoglobin: <7.5%; glucose (serum): 4.1–5.9 mmol/L; Insulin: 2–25 mcU/ml; HOMA IR (homeostatic model assessment for insulin resistance)): 0.23–2.5; HOMA β (homeostatic model assessment for insulin resistance for beta-cell function%): 0–100; urinary urea: 10–30 g/24 h; albumin (serum): 32–49 g/L; creatinine (serum): 50–120 mmol/L; 25(OH)D (Vitamin D, serum): 30–100 (ng/mL); PTH (Parathormone, serum): 10–70 pg/mL; Diastolic pressure: <90 mmHg; systolic pressure: <140 mmHg; p-value (chi squared test); PASE (Physical Activity Scale for the Elderly): 0–450.*

In women, significant differences emerged among the five clusters. In particular, the NW cluster shows the highest levels of HDL cholesterol, and the lowest ones of triglycerides, glycated hemoglobin, glucose, insulin, HOMA (IR and β), urea and diastolic pressure. On the contrary, the LOB cluster (with the highest BMI) have the lowest levels of calorie intake and HDL cholesterol and the highest ones of triglycerides, glycated hemoglobin, glucose, insulin, HOMA (IR and β), urea and diastolic pressure. No difference emerged for other parameters among the five clusters (Table 4A).

For each cluster, we also reported the number of participants using drugs for the control of cholesterol, glucose and blood pressure and supplementation of calcium and vitamin D_3_ that could impact on the metabolic profiles. Among the five BC clusters, no difference emerged in the number of women taking statins and drugs for the reduction of glycaemia, while hypertensive drugs were mostly used by the LOB cluster. This could explain the similar values in systolic blood pressure across the five clusters. It is interesting to note that the percentage of participants taking anti-hypertensive drugs is different between LOA and LOB (34.4% vs. 62.2%). Despite the similar BMI, the percentage of women taking anti-hypertensive drugs is higher in the cluster with higher fat parameters (Table 4A).

As for the physical functioning measures, LOA cluster has the highest values for the handgrip strength test (Table 4A). The LOA cluster is indeed characterized by the highest values for lean mass parameters (FMI, LMI, and ALMI) (Table 3A). The highest PASE score is found in the NW and the lowest one in the LOB cluster (Table 4A).

In men, participants within the NW cluster have the highest adherence to the NU-AGE diet and levels of HDL cholesterol and the lowest ones of triglycerides, glucose, insulin, HOMA IR and urea. On the contrary, the MO cluster (with the highest BMI) showed the lowest levels of calorie intake and HDL cholesterol and the highest ones of triglycerides, glucose, insulin, HOMA IR, HOMA β and urea. The OWA cluster has the lowest levels of HOMA β. The highest and the lowest levels of albumin are found within the LOA and LOB clusters, respectively, while the highest and the lowest levels of PTH are found within the OWB and OWA clusters, respectively. No difference emerged for other parameters (Table 4B).

**Table 4B:** Metabolic profile across the six body composition clusters in men.

	Normal weight	Overweight A	Overweight B	Low obesity A	Low obesity B	Moderate obesity	*p*-value
	(*n* = 122)	(*n* = 20)	(*n* = 233)	(*n* = 34)	(*n* = 80)	(*n* = 12)	
Adherence to NU-AGE diet	51.7 ± 9.2	50.9 ± 12.1	50.3 ± 9.1	49.2 ± 8.0	47.2 ± 9.7	49.5 ± 5.9	2.39e-02
Calorie intake (kcal)	2329.0 ± 425.3	2502.5 ± 390.2	2009.2 ± 408.9	2207.1 ± 458.7	2040.8 ± 446.3	1958.1 ± 244.4	7.538e-12
Cholesterol (mg/dL)	194.4 ± 33.6	195.2 ± 40.1	194.5 ± 39.3	189.5 ± 36.0	193.5 ± 41.8	199.8 ± 42.1	NS
HDL (mg/dL)	57.9 ± 16.1	53.3 ± 14.4	50.9 ± 13.7	50.1 ± 13.9	44.8 ± 12.1	40.6 ± 10.2	4.32e-09
LDL (mg/dL)	119.2 ± 30.3	121.6 ± 36.4	122.0 ± 35.4	117.4 ± 28.3	124.8 ± 36.5	132.7 ± 42.1	NS
Triglycerides (mg/dL)	86.3 ± 33.7	101.6 ± 46.9	108.0 ± 50.0	109.4 ± 49.8	120.3 ± 51.7	132.7 ± 36.2	6.95e-08
Glycated hemoglobin (%)	5.7 ± 0.4	5.7 ± 0.2	5.8 ± 0.5	5.8 ± 0.7	5.8 ± 0.7	5.9 ± 0.3	NS
Glucose (mmol/L)	5.6 ± 0.8	5.8 ± 0.9	5.9 ± 0.9	6.0 ± 1.1	6.1 ± 1.0	6.3 ± 0.7	3.78e-05
Insulin (mcU/mL)	6.3 ± 3.7	6.9 ± 2.7	9.6 ± 6.4	14.1 ± 14.1	14.1 ± 8.8	22.2 ± 10.7	<2.2e-16
HOMA IR	1.6 ± 1.1	1.8 ± 0.8	2.6 ± 2.0	3.9 ± 4.0	3.9 ± 2.6	6.4 ± 3.5	<2.2e-16
HOMA β (%)	67.4 ± 47.0	65.8 ± 24.8	85.8 ± 53.8	112.7 ± 98.7	117.4 ± 77.3	159.7 ± 76.8	1.42e-09
Urinary urea (g/24 h)	22.6 ± 6.0	24.0 ± 4.0	22.7 ± 6.1	25.5 ± 6.8	24.6 ± 7.6	31.6 ± 12.6	1.47e-03
Albumin (g/L)	45.9 ± 4.8	47.2 ± 4.8	45.4 ± 3.6	47.6 ± 4.5	44.3 ± 3.4	44.7 ± 3.5	4.832e-03
Creatinine (mmol/L)	88.6 ± 4.8	88.5 ± 17.2	90.3 ± 17.3	92.4 ± 17.2	89.2 ± 11.6	92.9 ± 27.4	NS
25(OH)D (ng/mL)	25.6 ± 8.7	24.7 ± 7.0	24.3 ± 8.5	23.4 ± 8.7	22.4 ± 8.4	25.2 ± 7.1	NS
PTH (pg/mL)	38.7 ± 27.1	40.0 ± 28.4	46.3 ± 22.5	37.1 ± 19.1	45.9 ± 22.0	46.1 ± 20.7	1.98e-03
Diastolic	77.4 ± 10.4	76.6 ± 8.2	76.2 ± 10.4	81.3 ± 7.6	77.7 ± 10.2	77.6 ± 8.1	NS
Systolic	134.9 ± 17.8	138.8 ± 15.2	138.8 ± 18.2	142.3 ± 16.7	142.7 ± 17.6	141.6 ± 16.9	NS
**Use of medicines/supplements**							
Statins (*n* = 130; %)	20.5	15.0	28.3	32.3	30.0	25.0	NS
Diabetics (*n* = 26; %)	3.2	0	10.7	2.9	11.3	8.3	NS
Hypertension (*n* = 242; %)	30.3	35.0	51.9	46.7	63.7	100.0	4.223e-08
Vitamin D (*n* = 26; %)	4.1	5.0	5.6	5.9	6.3	0.0	NS
Calcium (*n* = 15; %)	1.6	15.0	3.9	2.9	0.0	0.0	2.869e-02
**Physical functioning**							
Handgrip strength (kg)	39.6 ± 6.3	41.8 ± 7.5	38.2 ± 6.8	41.9 ± 7.9	41.3 ± 7.1	44.4 ± 9.1	5.653e-04
PASE score	153.8 ± 58.1	157.4 ± 65.1	139.3 ± 64.6	150.6 ± 65.2	125.2 ± 54.6	130.9 ± 61.2	2.895e-02

*Population based reference ranges for: Total Cholesterol: <200 mg/dL; HDL, (high density lipoprotein): >60 mg/dL; LDL (low density lipoprotein): <100 mg/dL; Triglycerides: <150 mg/d; glycated hemoglobin: <7.5%; Gglucose (serum): 4.1—5.9 mmol/L; Iinsulin: 2—25 mcU/ml; HOMA IR (Hhomeostatic model assessment for Iinsulin Rresistance)): 0.23—2.5; HOMA ((homeostatic model assessment for insulin resistance for beta-cell function%): 0–100; urinary urea: 10–30 g/24 h; Albumin (serum): 32–49 g/L; Creatinine (serum): 50–120 mmol/L; 25(OH)D (Vitamin D, serum): 30–100 (ng/mL); PTH (Parathormone, serum): 10–70 pg/mL; Diastolic pressure: <90 mmHg; Systolic pressure: (140 mmHg; p -value (chi squared test); PASE (Physical Activity Scale for the Elderly): 0—450.*

Among the six BC clusters identified in men, no difference emerged in the number of participants taking statins and drugs for the reduction of glycaemia, while all the participants within the LOB cluster used anti-hypertensive drugs. This could explain the similar values in systolic and diastolic blood pressure across the six clusters. It is interesting to note that the percentage of participants taking anti-hypertensive drugs is different between LOA and LOB (46.7% vs. 63.7%, respectively) and OWA and OWB (35.0% vs. 51.9%, respectively). Like for women, also for men the percentage of participants taking anti-hypertensive drugs is higher in the cluster with higher fat parameters. No difference emerged for the percentage of participants taking vitamin D supplementation with the exception of the MO cluster where no participant was taking vitamin D. Also, no participant within the LOB and MO was under calcium supplementation, while the 15% of participants within the OWA cluster used calcium supplementation. (Table 4B). Despite the fact that the levels of vitamin D [supplementation and also serum 25(OH)D level] were similar among the six clusters, OWA, LOB, and MO resulted to have higher values for bone mass parameters (*T*-score and BMC) (Table 3B).

As for the physical functioning measures, the LOA cluster has the highest values for the handgrip strength test (Table 4A). The LOA cluster is indeed characterized by the highest values for some lean mass parameters (LMI and ALMI) (Table 3B). The PASE score is highest in the OWA and lowest in the LOB cluster (Table 4B).

**FIGURE 1:**
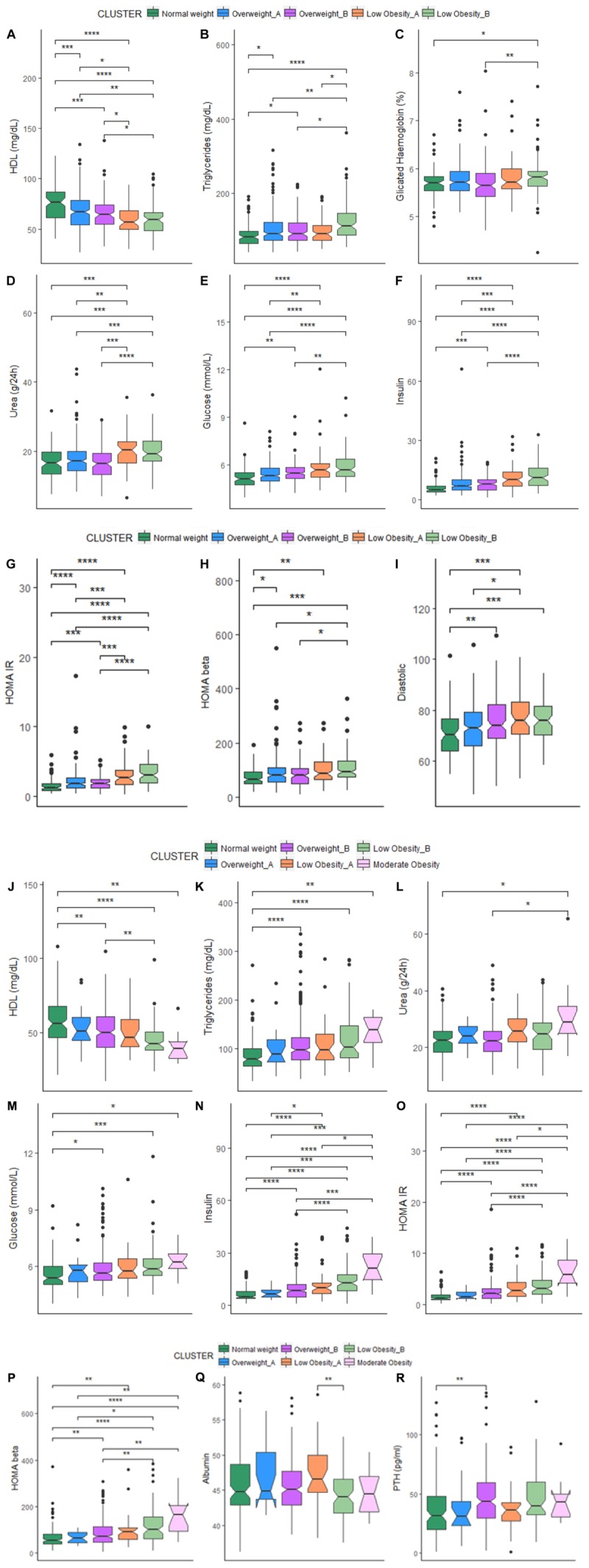
**(A–I)** Box-plots and significant differences of metabolic parameters among clusters in women. Statistical analysis was perfomed by Kruskal–Wallis test (*p*-values: ^∗^*p* < 0.05, ^∗∗^*p* < 0.01, ^∗∗∗^*p* < 0.001, ^∗∗∗∗^*p* < 0.0001). **(J–R)** Box-plots and significant differences of metabolic parameters among clusters in men. Statistical analysis was perfomed by Kruskal–Wallis test (*p*-values: ^∗^*p* < 0.05, ^∗∗^*p* < 0.01, ^∗∗∗^*p* < 0.001, ^∗∗∗∗^*p* < 0.0001).

The comparison of each metabolic parameter between each cluster is reported in Figure 1. When comparing cluster with similar BMI (OWA vs. OWB, LOA vs. LOB) no significant difference emerged for all the metabolic parameters, with the exception of triglycerides (Figure 1B) that resulted higher in women in LOB with respect to LOA; and values for albumin (Figure 1Q), that resulted higher in men in LOA with respect to LOB.

### Distribution of the BC Clusters Per Country

**FIGURE 2:**
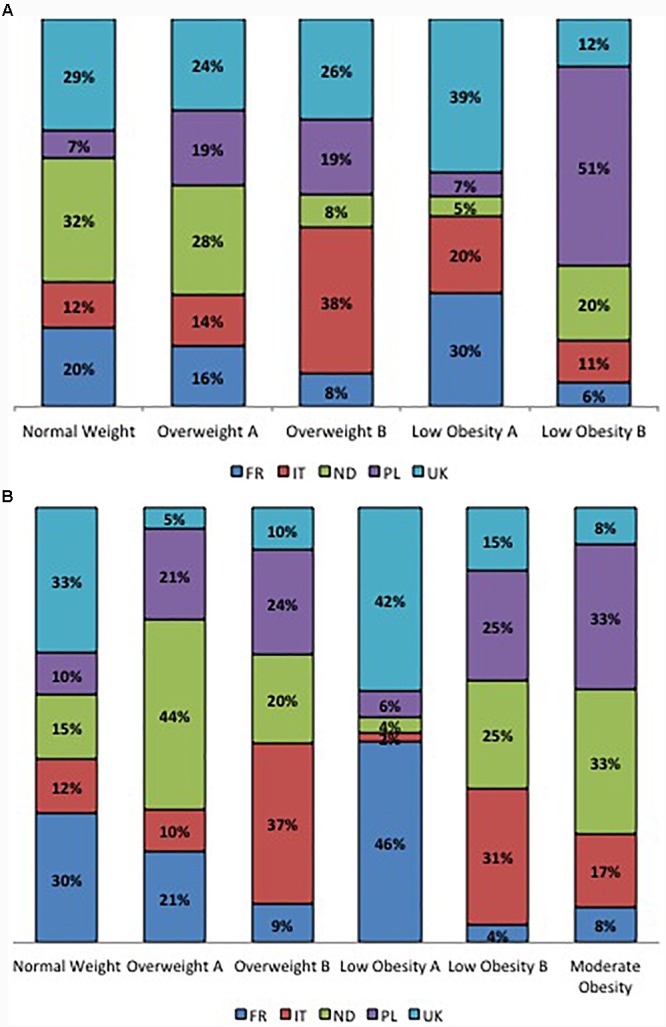
**(A)** Percentage contribution of the countries to clusters in women. **(B)** Percentage contribution of the countries to clusters in men.

The percentage of participants within each BC cluster is reported in Figure 2 considering sex and country of origin.

In women, within the NW and the OWA clusters, the majority of participants comes from the Netherlands (32 and 28%, respectively) and United Kingdom (29 and 24%, respectively); the OWB cluster is mainly represented by the Italians (38%) followed by the English participants (26%); the LOA cluster is mainly represented by English (39%) and French participants (30%) while the LOB cluster is represented for the 51% by Polish participants followed by a 20% of Dutch participants (Figure 2A).

In men, the NW cluster is mainly composed by English (33%) and French (30%) participants while the OWA cluster is mainly composed by Dutch (44%) Polish (21%) and French (21%) participants, within the OWB cluster the majority of participants belong to Italy (37%) and Poland (24%), the majority of English (42%) and French (46%) participants belong to the LOA cluster, the LOB cluster is mainly composed by Italians (31%), Dutch (25%) and Polish (25%), the MO cluster is mainly composed by Dutch (33%) and Polish (33%) participants equally (Figure 2B).

## Discussion

The aim of this study is to provide a snapshot of BC in the elderly across Europe, taking advantage of the data on fat, lean and bone mass evaluated by DXA scan on 1121 elderly participants to the European project NU-AGE.

The first evidence emerging from this study is the persistence also in old age of sex-specific difference in BC, despite the fact that sex hormones, which are very important determinants of BC, tend to decrease with age in both sexes. However, sex differences in terms of rate of age-related loss of muscle and bone mass, as well as deposition of fat are reported (Lauretta et al., 2017). Therefore this finding was not surprising and all the subsequent analyses were performed separately for men and women.

Beside the sex differences, our results also showed a marked difference in BC among the 5 countries where NU-AGE participants were recruited. On the whole, French participants were thinner compared to participants from other countries. They have the lowest values for FM parameters, weight and anthropometric measures, and the highest ones for lean mass parameters. On the contrary Polish participants have the highest values for FM and Dutch have the highest bone mass parameters. These differences are not explained by enrollment bias, as inclusion and exclusion criteria for the NU-AGE participants were identical in the five recruiting countries (Santoro et al., 2014). These differences in BC could be rather ascribed to lifestyle and genetic characteristics of the different populations. BC is indeed the result of the genetic predisposition and of the lifelong dietary and physical activity habits even if many other modulators such as environmental structures, cultural values, economic factors, education, stress among others can contribute. In this study, the level of education seems surprisingly not correlated with good dietary habits, as it would be expected (Schoufour et al., 2017). In fact, participants from Poland result the most educated with a total number of school years of 15.4 and most of them (77.5%) attended the college; however, they are the most robust and have the lowest knowledge on nutritional values (Jeruszka-Bielak et al., 2018). French participants at variance have an average of 12.5 school years and they resulted the thinnest and the most adherent to the NU-AGE diet (55.9). Dutch participants have a very similar number of school years (12.3), but they have lowest (46.3) adherence to the NU-AGE diet. This suggests that cultural habits as well as food traditions overcome education in modulating BC. In fact, the French cohort has the higher calorie intake, but also a higher intake of fish and low fat meat and poultry (Berendsen et al., unpublished) that likely account for a high protein intake and could contribute to the higher level of lean mass. While Polish people have higher intakes of whole grains, eggs, vegetable and low fat cheese and salt maybe contributing to the high fat levels (Berendsen et al., unpublished). However, despite the highest values for lean mass French elderly do not show the highest values for physical activity neither for PASE score nor for the handgrip strength while Polish people showed the lowest values for the handgrip strength, but not for the PASE score.

It is known that there is variation in the prevalence of specific alleles between the north and south of Europe. Certain aspects of obesity represent heritable traits, with heritability estimates varying between 40 and 70% depending on the populations examined. Approximately 128 alleles have been associated with some parameters related to obesity (visceral fat, waist circumference, insulin resistance etc.). Possession of the predisposing alleles does not constitute a biological inevitability for the development of obesity, and it has been demonstrated that the influence of certain alleles can be counteracted by a high level of physical activity (Vimaleswaran et al., 2009) and diet (Corella and Ordovás, 2015). Taken together, it is likely that the operation of any genetic factor can be affected or masked by environmental effects (Ly and Loos, 2013). Epigenetics and its variable effects on numerous phenomena add further complexity. Given the intimate association between food consumption and body weight, it is essential to consider whether food availability or some factor in the food culture could determine the level of obesity in a particular country. There is considerable evidence that exposure to particular types of foods or diets is associated with the over- consumption of energy and is a risk factor for weight gain. There is also evidence that portion size exerts an influence on the daily energy consumed. Other evidence draws attention to the snacking habit, i.e., the uncompensated eating events between meals (Blundell et al., 2017).

It is well known that in adult population (18–75 years), the rates of obesity in Europe are very different among countries with the lowest rates in Romania (9.4%), Italy (10.7%), Netherlands (13.3%), Belgium and Sweden (14.0% both) and the highest in Malta (26%), Latvia (21.3%), Hungary (21.2%) Estonia (20.4%), and United Kingdom (20.1%) (EUROSTAT, 2016). When stratifying by age group, the presence of obesity in elderly persons aged 65–74 years among the five EU countries represented in this study are 22.5% for France, 15.7% for Italy, 17.7% for Netherlands, 28.4% for Poland and 20.7% for United Kingdom (EUROSTAT, 2016). Our results are only in part supporting these data, as participants from Poland had higher fat parameters than the others, but elderly from France resulted the slimmest in contrast with data from Eurostat. These differences could be explained by the fact that, even if the data coming from the Eurostat are from a large survey, they are based only on self reported BMIs while data on the NU-AGE population are obtained from accurate and standardized measures (anthropometry and DXA). Moreover, a partial bias of recruitment cannot be excluded for the NU-AGE study, as it was based on volunteers that may not perfectly reflect the whole European population.

Although BMI is the most frequently used index of obesity, it fails to account for BC and to distinguish between the relative contribution of FM and lean mass (Blundell et al., 2014), the latter declining with age. This can further lead to misclassification and potential underestimation of adiposity. In this study we used a cluster analysis to verify the overlapping between BMI classification and BC in elderly. Our results identified clusters with similar BMI, but different fat, lean and bone mass composition in both sexes, demonstrating that BMI is not a suitable tool to discriminate between body fat, lean and bone mass content and distribution (trunk, limbs etc.). It is now clearly recognized that BMI is a polygenic trait and more than 700 genes are involved in the modulation of body height (used in the calculation of BMI). Population- genetic differences in BMI were recently detected amongst almost 10,000 individuals across 14 European countries. Approximately 8% (95% CI 4–16%) of the captured additive genetic variance for BMI reflected population-genetic differences.

In this study we have compared a series of metabolic parameters across the identified BC clusters. It is indeed known that accumulation of fat is a risk factor for increased morbidity, impaired quality of life, and premature death. As a whole, the NU-AGE study participants are healthy participants and, as expected, all metabolic parameters analyzed are within the normal range both in men and women, however, significant differences exist among the clusters. In particular, it is interesting to note that these differences are found not only when comparing clusters with different BMI, but also between clusters with very similar BMI, indicating that the sole indication of BMI can lead to a misclassification of participants into wrong risk groups.

The number of participants taking drugs for the control of cholesterol (statins) does not differ among the clusters and this could explain the similar values in cholesterol and LDL levels across the clusters both in men and women. The majority of elderly taking anti-hypertensive drugs belongs to the LOB cluster. It is interesting to note that the percentage of participants taking anti-hypertensive drugs is higher in LOB compared with LOA. Despite the similar BMI the percentage of elderly taking anti-hypertensive drugs is higher in the cluster with higher fat parameters. The measured levels of circulating 25(OH)D resulted not different among the clusters, however, it is interesting to note that the majority of elderly taking vitamin D and calcium supplementation is found within the clusters with higher BMI (OWA, OWB, LOB, and MO) and have also the highest values for bone mass parameters (*T*-score and BMC).

Finally, elderly within the LOA cluster have the highest values for the handgrip strength test and are characterized by the highest values for lean mass parameters (FMI, LMI, and ALMI). It is to note that an obesity paradox exists, as apparently an increased body weight has protective effects, being associated with decreased mortality in selected populations. This paradox could be explained by the inability of BMI to discern between visceral and subcutaneous fat. Another possible explanation is the pleiotropy of adiposity. Certainly excess adiposity is a risk factor for a great number of pathologies, however, it could be that a certain degree of adiposity may be required for longevity (Batsis and Zagaria, 2018), and in this perspective it may make sense to find better functional parameters associated with adiposity in elderly people. Moreover, although fall risk is increased, risk of hip fractures related to obesity is lower in certain populations (Tang et al., 2013; Fassio et al., 2018). As such, practitioners should be made aware of these considerations, particularly when using BMI as a measure for adiposity in older patients.

Some weakness are present in our study: the NU-AGE participants are in fact apparently healthy volunteers interested in nutrition and health topics and they are also highly educated with respect to the average of the general population of the same age-cohort thus they could be not totally representative of the European general elderly population. Another major limitation of our study regards the lack of a cross-calibration among the different DXA scanners used, thus the comparison among the raw BMD and BMC should be kept with caution as suggested by the ISCD guidelines (International Society for Clinical Densitometry, 2015). Moreover, we cannot exclude that also the comparison of fat and lean mass measures across countries could be affected by systematic differences. Several studies have indeed shown that the use of machines from different manufacturers also impinges upon the comparison of fat and lean mass measures (Fan et al., 2013; Krueger et al., 2016) and a consensus for this correction has not been found yet (International Society for Clinical Densitometry, 2015). However, despite the use of different DXA scanners impact on the variance of the measures among the five countries, and should be considered another limitation of the study, the ability to study BC by DXA at a European level is a unique feature and important strength of the NU-AGE project. The use of standardized methodology, indexes and methods without relying on self reported data is also another strength of this study.

In conclusion, the results presented in this paper represent a report on the BC status of healthy elderly men and women in Europe, to be used as a reference for future investigations on pathological conditions and differences between countries.

## Author Contributions

AS, AlB, and CF contributed to the conception, design and data interpretation of the current work. AS drafted the manuscript. GG, EG, and DM contributed to data analyses. CF conceived, designed, initiated and directed NU-AGE. AS coordinated NU-AGE data collection across centers. AgB and LDG designed the dietary intervention study. RO, MS, OS, AJ, NM, EC, RG, BP, GB, FK, FC, KC, and SS substantially contributed to the data collection by acquiring or processing data. All authors contributed to interpretation of data, critically revised and approved the final version of this manuscript.

## Conflict of Interest Statement

The authors declare that the research was conducted in the absence of any commercial or financial relationships that could be construed as a potential conflict of interest.

## Footnotes

1 http://www.nu-age.eu/

## Supplementary Material

FIGURE S1 **(A–F)** Box-plots of participants’ anthropometric characteristics divided by sex and country, and significant differences among countries in women and men. Statistical analysis was perfomed by Kruskal–Wallis test (*p*-values: ^∗^*p* < 0.05, ^∗∗^*p* < 0.01, ^∗∗∗^*p* < 0.001, ^∗∗∗∗^*p* < 0.0001).

FIGURE S2 **(A,B)** Box-plots of participants’ diet assessment divided by sex and country, and significant differences among countries in women and men. Statistical analysis was perfomed by Kruskal–Wallis test (*p*-values: ^∗^*p* < 0.05, ^∗∗^*p* < 0.01, ^∗∗∗^*p* < 0.001, ^∗∗∗∗^*p* < 0.0001).

FIGURE S3 **(A,B)** Box-plots of participants’ Physical Funcitioning divided by sex and country, and significant differences among countries in women and men. Statistical analysis was perfomed by Kruskal–Wallis test (*p*-values: ^∗^*p* < 0.05, ^∗∗^*p* < 0.01, ^∗∗∗^*p* < 0.001, ^∗∗∗∗^*p* < 0.0001).

FIGURE S4 **(A–O)** Box-plots of participants’ Body Composition parameters divided by sex and country, and significant differences among countries in women and men. Statistical analysis was perfomed by Kruskal–Wallis test (*p*-values: ^∗^*p* < 0.05, ^∗∗^*p* < 0.01, ^∗∗∗^*p* < 0.001, ^∗∗∗∗^*p* < 0.0001).
