# Supplementary figures and images for: A Cross-Sectional Analysis of Body Composition Among Healthy Elderly From the European NU-AGE Study: Sex and Country Specific Features
Source: Front Physiol. 2018 Nov 30;9:1693. doi: 10.3389/fphys.2018.01693 (PMC6283977; doi:10.3389/fphys.2018.01693)

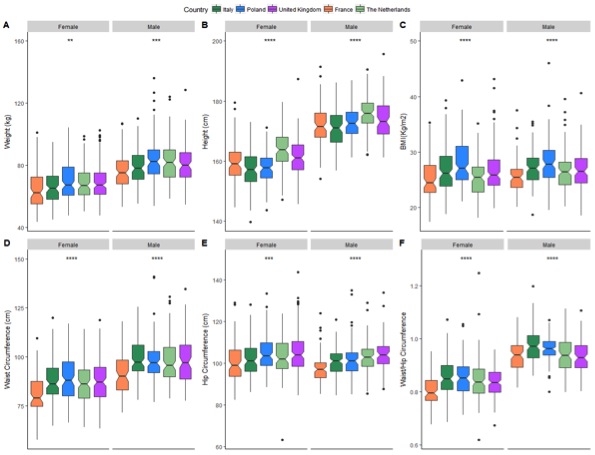

Supplement: FIGURE S1 — (A–F) Box-plots of participants’ anthropometric characteristics divided by sex and country, and significant differences among countries in women and men. Statistical analysis was perfomed by Kruskal–Wallis test (p-values: ∗p < 0.05, ∗∗p < 0.01, ∗∗∗p < 0.001, ∗∗∗∗p < 0.0001). [file Image_1.JPEG]

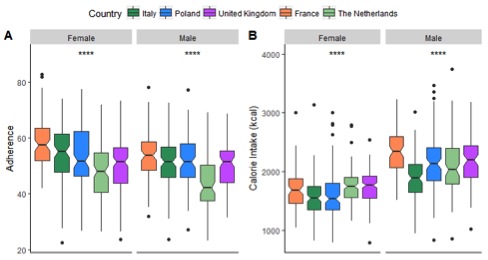

Supplement: FIGURE S2 — (A,B) Box-plots of participants’ diet assessment divided by sex and country, and significant differences among countries in women and men. Statistical analysis was perfomed by Kruskal–Wallis test (p-values: ∗p < 0.05, ∗∗p < 0.01, ∗∗∗p < 0.001, ∗∗∗∗p < 0.0001). [file Image_2.JPEG]

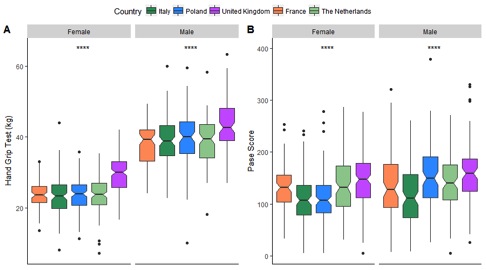

Supplement: FIGURE S3 — (A,B) Box-plots of participants’ Physical Funcitioning divided by sex and country, and significant differences among countries in women and men. Statistical analysis was perfomed by Kruskal–Wallis test (p-values: ∗p < 0.05, ∗∗p < 0.01, ∗∗∗p < 0.001, ∗∗∗∗p < 0.0001). [file Image_3.JPEG]

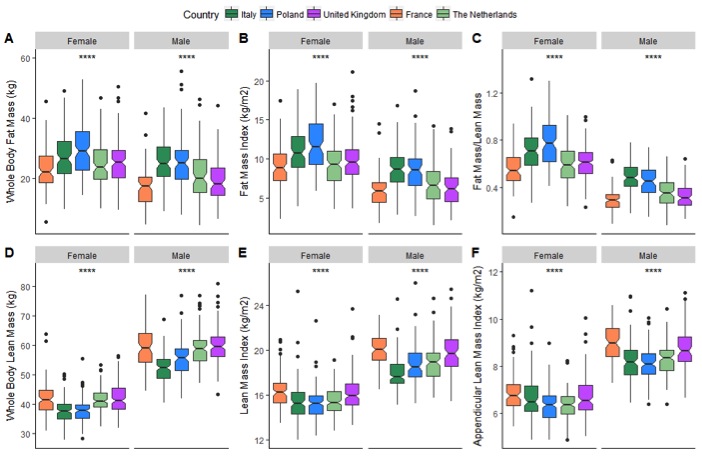

Supplement: FIGURE S4 — (A–O) Box-plots of participants’ Body Composition parameters divided by sex and country, and significant differences among countries in women and men. Statistical analysis was perfomed by Kruskal–Wallis test (p-values: ∗p < 0.05, ∗∗p < 0.01, ∗∗∗p < 0.001, ∗∗∗∗p < 0.0001). [file Image_4.JPEG]

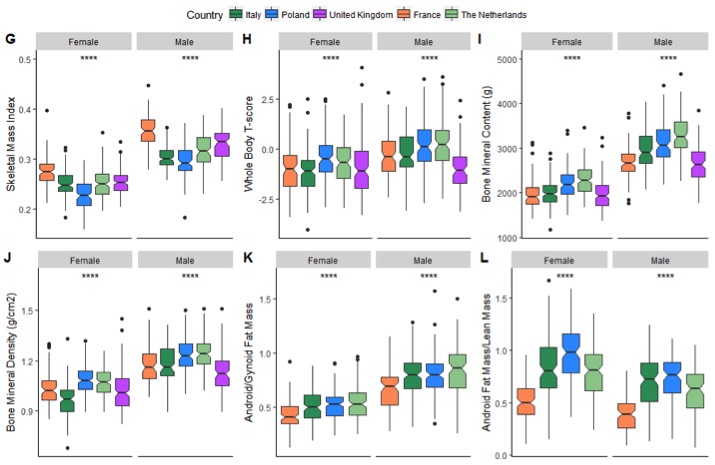

Supplement: Supplementary file 5 [file Image_5.JPEG]

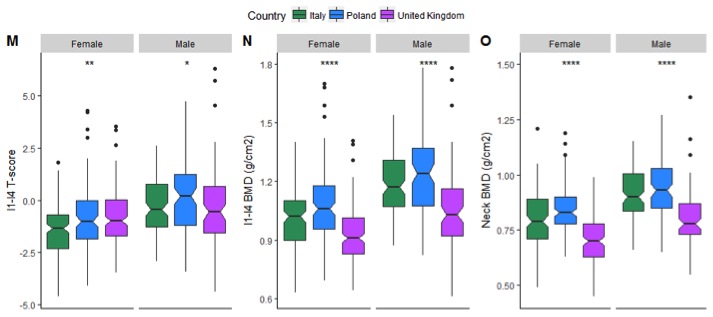

Supplement: Supplementary file 6 [file Image_6.JPEG]
